# Deep Learning With Optical Coherence Tomography for Melanoma Identification and Risk Prediction

**DOI:** 10.1002/jbio.202400277

**Published:** 2024-10-27

**Authors:** Pei‐Yu Lai, Tai‐Yu Shih, Yu‐Huan Chang, Chung‐Hsing Chang, Wen‐Chuan Kuo

**Affiliations:** ^1^ Institute of Biophotonics National Yang Ming Chiao Tung University Taipei Taiwan; ^2^ Hualien Tzu chi Hospital, Buddhist Tzu Chi Medical Foundation Skin Institute Hualien Taiwan; ^3^ Doctoral Degree Program in Translational Medicine Tzu chi University and Academia Sinica Hualien Taiwan; ^4^ Institute of Medical Sciences Tzu chi University Hualien Taiwan

**Keywords:** convolutional neural network, melanoma, mice model, optical coherence tomography, risk prediction

## Abstract

Malignant melanoma is the most severe skin cancer with a rising incidence rate. Several noninvasive image techniques and computer‐aided diagnosis systems have been developed to help find melanoma in its early stages. However, most previous research utilized dermoscopic images to build a diagnosis model, and only a few used prospective datasets. This study develops and evaluates a convolutional neural network (CNN) for melanoma identification and risk prediction using optical coherence tomography (OCT) imaging of mice skin. Longitudinal tests are performed on four animal models: melanoma mice, dysplastic nevus mice, and their respective controls. The CNN classifies melanoma and healthy tissues with high sensitivity (0.99) and specificity (0.98) and also assigns a risk score to each image based on the probability of melanoma presence, which may facilitate early diagnosis and management of melanoma in clinical settings.

## Graphical abstract

This study uses the noninvasive optical coherence tomography (OCT) technique to add a novel and practical approach to melanoma diagnosis. In addition, the convolutional neural network (CNN) model can provide a risk score for each OCT image, indicating the probability of melanoma presence.

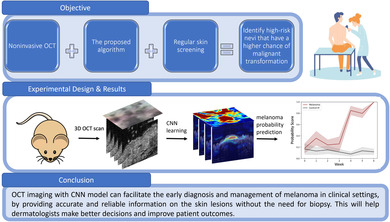

## 1 Introduction

Cutaneous melanoma originates in melanocytes and is the most dangerous type of skin cancer. More than half of the melanomas develop from nevi in young patients, and nevus‐associated melanomas are approximately 33% in all age groups [1, 2]. Therefore, detecting and removing the nevus with a high probability of transformation to melanoma may be useful and reduce melanoma mortality rates. Moreover, the majority of melanoma patients are diagnosed with T1 (1 mm) tumors. The mortality rate for patients with thin primary melanomas (T1) is higher than those with thick primary melanomas [3]. Therefore, early detection of melanoma “in situ” and follow‐up management improve patient survival. However, differentiating between nevus and melanoma by histopathological characteristics can be difficult, and discordance in the diagnosis among dermatopathologists has been reported in previous studies [4, 5, 6]. Besides, the biopsy procedure is invasive, time‐consuming, and costly. Therefore, there is still a need to develop noninvasive imaging techniques to acquire sequential datasets, which may also help understand the transition from benign lesions to melanoma and improve early diagnosis.

For the above reasons, many studies focus on developing noninvasive imaging techniques to aid melanoma diagnosis. Reflectance confocal microscopy (RCM), high‐frequency ultrasound (HFUS), photoacoustic imaging (PAI), and optical coherence tomography (OCT) are emerging techniques for the diagnosis of melanoma. The RCM has a resolution comparable to histology and can provide cellular features of melanoma that correlate well with histopathological findings [7]. However, the shallow penetration depth of ~250 μm limits the observation to the upper reticular dermis or the papillary dermis [8]. HFUS is used mainly to measure the thickness of the melanoma and is less used in diagnosing melanoma due to poor contrast and lack of tumor‐specific characteristics [9]. PAI uses the absorbing property as an endogenous contrast agent for melanoma imaging. Still, PAI is used mainly to assess the thickness, and skin pigmentation may affect the diagnosis of melanoma [10].

Previous studies have reported specific characteristics of melanoma in conventional OCT images, such as more significant architectural disorganization, less definition, and absence of a lower border of the lesions compared to benign nevi [11, 12] and associated histopathological characteristics in high‐definition (HD) OCT [13, 14, 15]. Although OCT cannot be used to diagnose more advanced melanoma due to the shallow penetration depth (i.e., 1–2 mm), it may be helpful for risk prediction in early melanoma. By quantifying in vivo optical properties such as light attenuation in melanocytic lesions by HD‐OCT, Boone et al. [13] reported a sensitivity of 93.3% and a specificity of 96.7% of HD‐OCT for the differentiation of melanoma from nonmalignant lesions. Zahra et al. use optical radiomic signatures derived from OCT images: the mean and standard deviation of the scattering coefficient, the absorption coefficient, and the anisotropy factor to differentiate benign nevi and melanoma with a sensitivity of 97% and a specificity of 98% [16]. However, the generalizability of radiomics models is still challenging, limiting their implementation into clinical practice [17].

On the other hand, previous studies have shown that deep learning (DL) can improve the accuracy of melanoma diagnosis, leading to early detection without requiring many invasive biopsy procedures [18, 19]. However, these studies mainly concentrated on dermoscopic images and ignored evolutionary features. Our study evaluated a DL model against previous methods [13, 14, 15, 16, 17]. This is the first study to use DL on OCT cross‐sectional images for separating melanoma from normal tissue while also considering melanoma progression and training models with prospective data. We validated the trained model using both melanoma and dysplastic nevus samples. Moreover, the algorithm network is analyzed to understand what it learns.

## 2 Materials and Methods

### 2.1 OCT System Setup

This study uses a spectral domain (SD) OCT system developed in‐house with a central wavelength of 1275 nm and a spectral bandwidth of 240 nm [20]. The system delivers an axial and lateral resolution of approximately 5 μm and 7 μm, respectively. It takes 50 s to obtain a 3D volume, and there are 400 cross‐sectional images in one 3D volume (4 × 4 × 2 mm^3^).

### 2.2 Mouse Model

**FIGURE 1:**
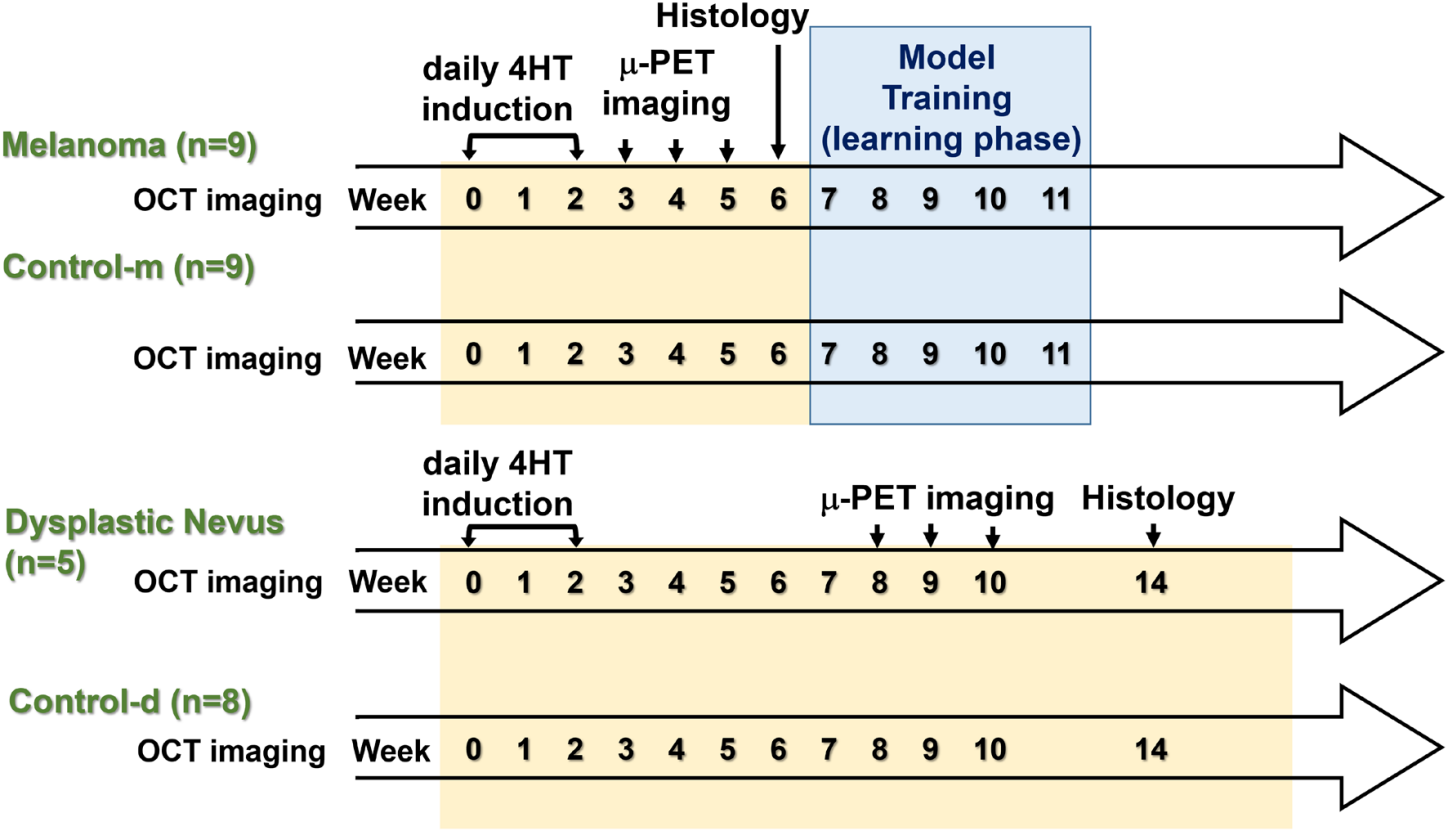
Schematic of the experimental timeline.

A breeding pair of B6.Cg‐Braftm1MmcmPtentm1HwuTg (Tyr‐Cre/ERT2)13Bos/BosJ mice (stock #013590) was purchased from the Jackson Laboratory. This experiment has four groups, as shown in Table 1. In the melanoma and dysplastic nevus mice group, 4‐Hydroxytamoxifen (4‐HT) was topically treated in the dorsal part of ears 10 times over 2 weeks to induce BRAF^V600E^ and silence PTEN expression, resulting in rapid melanoma development with significant penetrance and metastasis [21, 22]. The PTEN tumor suppressor is partially lost in dysplastic nevus mice and completely lost in melanoma mice. In the control‐m and control‐d groups, transgenic mice were not treated with 4‐HT. The schematic of the experiment is shown in Figure 1. Serial OCT images were collected every week from the ears of melanoma mice (*n* = 9), dysplastic nevi mice (*n* = 5), control‐m (*n* = 9), and control‐d mice (*n* = 8). Areas with less hair and areas that were easier to fix on the stage were selected for OCT imaging to reduce both image artifacts and motion effects. Week 0 refers to when the mice had not been treated with 4‐HT. After 7 days, the time point is set as week 1, etc. The μPET images (FLEX Triumph Pre‐Clinical Imaging System) were obtained at weeks 3, 4, and 5 in melanoma mice to confirm the timing of metastasis of melanoma and at weeks 8, 9, and 10 in dysplastic nevus mice to verify the transformation of dysplastic nevi into malignant melanoma. The histology (tissue analysis) of the melanoma mice (week 6) and dysplastic nevus mice (week 14) was obtained to confirm the diagnosis of metastasis and melanoma. The Institutional Animal Care and Use Committee (IACUC) of the National Yang Ming Chiao Tung University reviewed and approved the animal study.

**TABLE 1:** Mouse groups in the study.

Group	Braf	Pten	4‐HT
Melanoma	f/f	f/f	v
Melanoma control (control‐m)	f/f	f/f	x
Dysplastic nevus	f/f	f/+	v
Dysplastic nevus control (control‐d)	f/f	f/+	x

### 2.3 Datasets

Images collected from melanoma mice and control‐m after week 6 (i.e., weeks 7–11) are used in the learning phase, with 20 390 images for the induced group and 11 616 for the control group. Five‐fold cross‐validation was performed to evaluate the proposed method. One‐fold was selected as the testing set, and the remaining data was split into training and validation sets with a 9:1 ratio. Only the training and validation sets were used to modify the hyperparameters and select the model during the training process. No image of the same mouse appears in the training and testing sets. The trained model was used to predict the melanoma probability scores in melanoma mice and control‐m mice from weeks 0 to 6 to verify if the trained model could detect melanoma early. Furthermore, the same procedure was performed in dysplastic nevus mice and control‐d mice from weeks 0 to 14.

### 2.4 Attenuation Coefficient Analysis

The attenuation coefficient (AC) was obtained by fitting the scattering signal along the depth of each A‐line in the OCT cross‐sectional image. The depth range was chosen at 200 μm below the skin surface, covering the dermal–epidermal junction in the ears of mice. The training and validation dataset is used to select the optical attenuation threshold, and the test dataset is used to test and evaluate the identification result based on the threshold.

### 2.5 Radiomic Features Combined With Machine Learning Classifiers

The extraction of radiomics features, including first‐order statistical features and texture features, is performed in two regions (melanoma and control) using Pyradiomics packages [23]. Since the superficial spreading melanoma has an undefined border in the OCT image and is invisible to the naked eye, the tumor region was selected 200 μm below the skin surface, covering the dermal‐epidermal junction in melanoma mice ears. The normal region was selected at 200 μm below the skin surface in the ears of control‐m mice. The top five features include kurtosis, the maximum probability of the gray‐level co‐occurrence matrix (GLCM) matrix, joint energy of the GLCM matrix, interquartile range, and robust mean absolute deviation are selected according to the t‐test and minimum redundancy maximum relevance (mRmR) [24]. Decision trees were selected as the classifier according to the area under curve (AUC) value.

### 2.6 Proposed Architecture

**FIGURE 2:**
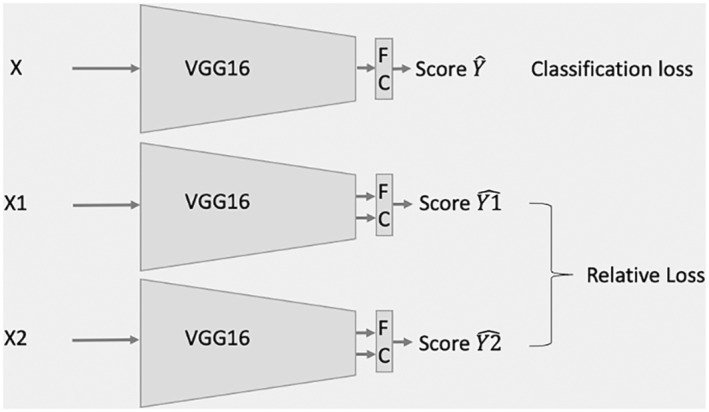
Illustration of the convolutional neural network (CNN) architecture.

A DL system was built for binary classification (differentiating cancerous OCT cross‐sectional images from normal ones). We used VGG16 [25] as the backbone, modified the last fully connected layer, and set its output dimensions to one. Here, the single‐channel OCT intensity images were replicated into three channels. The depth range in each OCT image was reduced from 1024 pixels to 512 pixels since the signal is typically present within this 512 pixel range. The lateral dimension was downsampled from 813 to 512 pixels to maintain symmetry and fit the VGG model. Each cross‐sectional OCT image is used as an individual input with no overlap. Thus, the model receives an OCT image, X, sized 512 × 512 × 3. The output consists of melanoma probability scores between 0 and 1. The network was trained using binary cross‐entropy loss, denoted as *Loss*_*BCE*_. In addition to binary cross‐entropy loss, we leveraged the sequential data and added another loss term, relative loss, $\mathrm{Los}s_{R}$. As shown in Figure 2, X1 and X2 are OCT cross‐sectional paired images of the same lesion, but they are taken at different time points. X1 was taken before X2. The time interval is more than 2 weeks in each pair. The prediction scores for X1 and X2 are denoted as $\hat{Y1}$ and $\hat{Y2}$, respectively. The relative loss $\mathrm{Los}s_{R}$forces that the output score at the later time point ($\hat{Y2})$ to be higher than at the earlier time point ($\hat{Y1}),$as shown in Equation (1), where *N* represents the number of images.

(1) $$\;\mathrm{Los}s_{R}=\frac{1}{N}\sum_{i=1}^{N}\text{ReLu}\left({\hat{\;Y1}}_{i}-{\hat{Y2}}_{i}\right)$$

The weighting is initialized with ImageNet pre‐trained weights, and the model is trained with $\mathrm{Los}s_{\mathrm{BCE}}$ and $\mathrm{Los}s_{R}$ (a ratio of 1:1) with Adam Optimizer. This framework is implemented with PyTorch and is trained with a GeForce RTX 1080 Ti graphics card. The training is stopped when the accuracy of the validation set does not improve for five epochs.

## 3 Results

**FIGURE 3:**
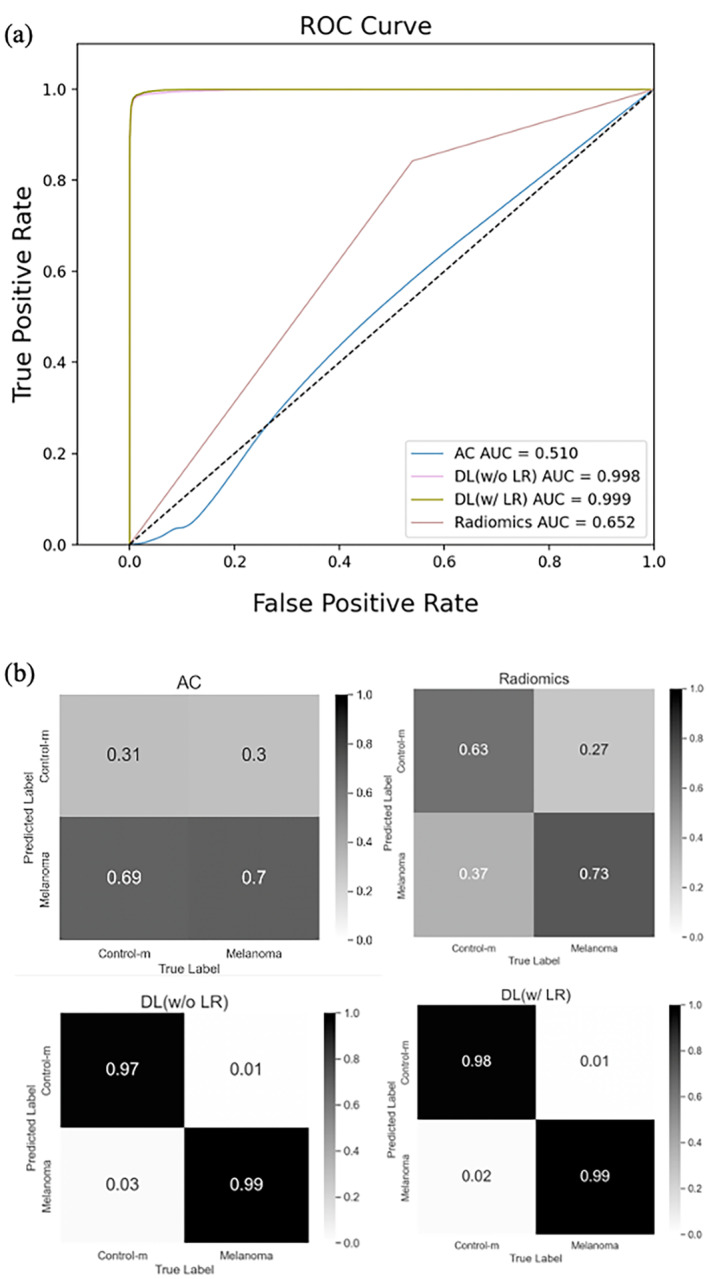
(a) The ROC curves and (b) the confusion matrices for the attenuation coefficient model, radiomics model, and deep learning model training without $\mathrm{Los}s_{R}$ and deep learning model training with $\mathrm{Los}s_{R}$. AC: Attenuation coefficient; DL: Deep learning. The AC model has an accuracy, sensitivity, and specificity of 0.50, 0.70, and 0.31, respectively. The radiomics model has an AUC of 0.65, accuracy of 0.68, sensitivity of 0.73, and specificity of 0.63. The deep learning model trained without $\mathrm{Los}s_{R}$ has an AUC of 0.998, an accuracy of 0.98, a sensitivity of 0.99, and a specificity of 0.97. The DL model trained with $\mathrm{Los}s_{R}$ has an AUC of 0.999, accuracy of 0.985, sensitivity of 0.99, and specificity of 0.98.

**FIGURE 4:**
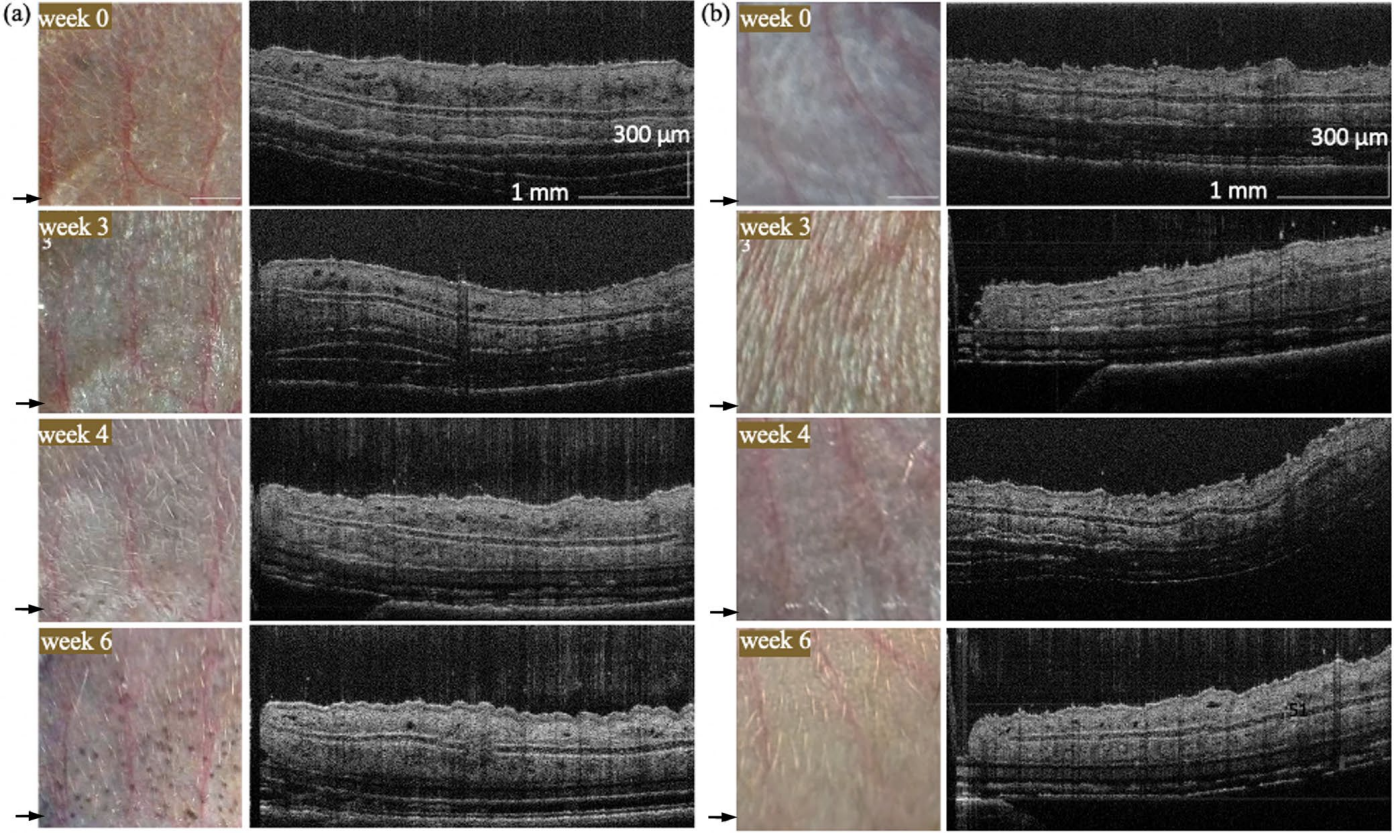
Photograph and corresponding cross‐sectional image of (a) melanoma and (b) control‐m mice at weeks 0, 3, 4, and 6. Arrows indicate the location corresponding to the cross‐sectional image.

**FIGURE 5:**
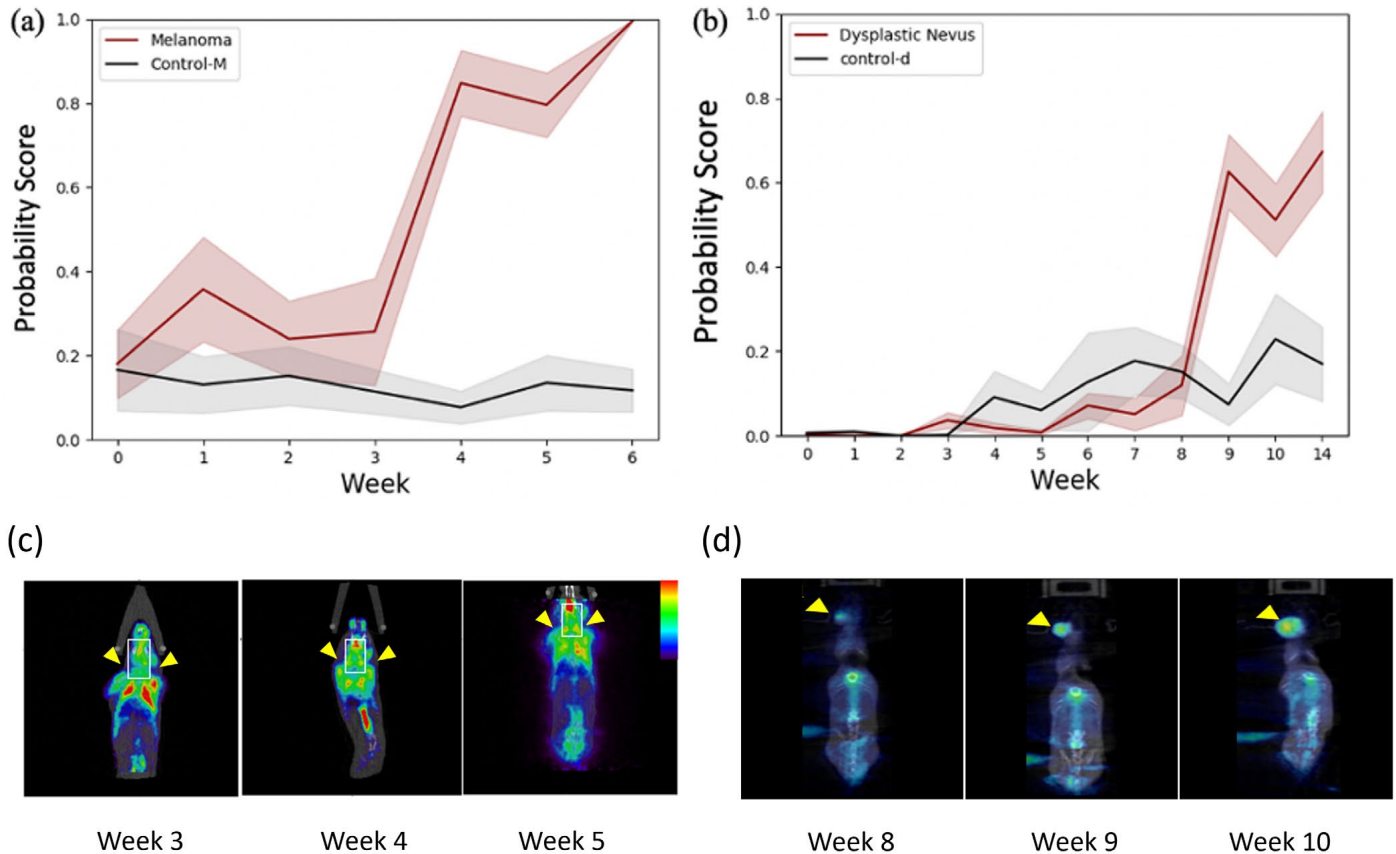
Melanoma probability scores of (a) melanoma (*n* = 9) and control‐m (n = 9) mice and (b) dysplastic nevus (*n* = 5) and control‐d (*n* = 8) mice. Longitudinal 18 F‐FDG μPET/CT images of (c) Melanoma mice and (d) dysplastic nevus mice.

Figure 3a shows the receiver operating characteristic (ROC) curves for different classifiers. The AC model has an AUC of 0.51, indicating similarity to a random guess. Confusion matrices in Figure 4b demonstrate the classification performance for the AC model, radiomics model, and DL model training without $\mathrm{Los}s_{R}$ and with $\mathrm{Los}s_{R}$. Incorporating relative loss in training aligns closely with human diagnostic practices, often involving assessing changes over time to make more accurate evaluations. Despite the lack of a significant performance boost in our study, the nuanced guidance provided by relative loss remains a valuable aspect of our model's development. After the learning stage, the trained model was used to predict the melanoma probability score of melanoma and control‐m mice from weeks 0 to 6; note that these OCT cross‐sectional images were not used in the learning phase. Figure 4 shows the photograph and the corresponding OCT cross‐sectional images. After induction of 4‐HT after week 0, the BRAF^V600E^ mutation and loss of PTEN induce melanocyte proliferation in melanoma mice (Figure 4a), while the appearance of the ear in control‐m mice remains the same at different time points (Figure 4b). There are no apparent tumors on the skin of melanoma mice in week 4, and not so much abnormality can be observed in the OCT cross‐sectional image by the naked eye. However, the melanoma probability score increases after week 3 and remains high after week 4, as shown in Figure 5a. The melanoma probability score is the average from 400 slices in 3D volume, indicating the percentage of malignancy in the scanned area. The μPET images (Figure 5c) showed increased uptake of 18F‐FDG (a radioactive tracer) in the cervical lymph nodes of melanoma mice at weeks 4 and 5, indicating metastasis.

**FIGURE 6:**
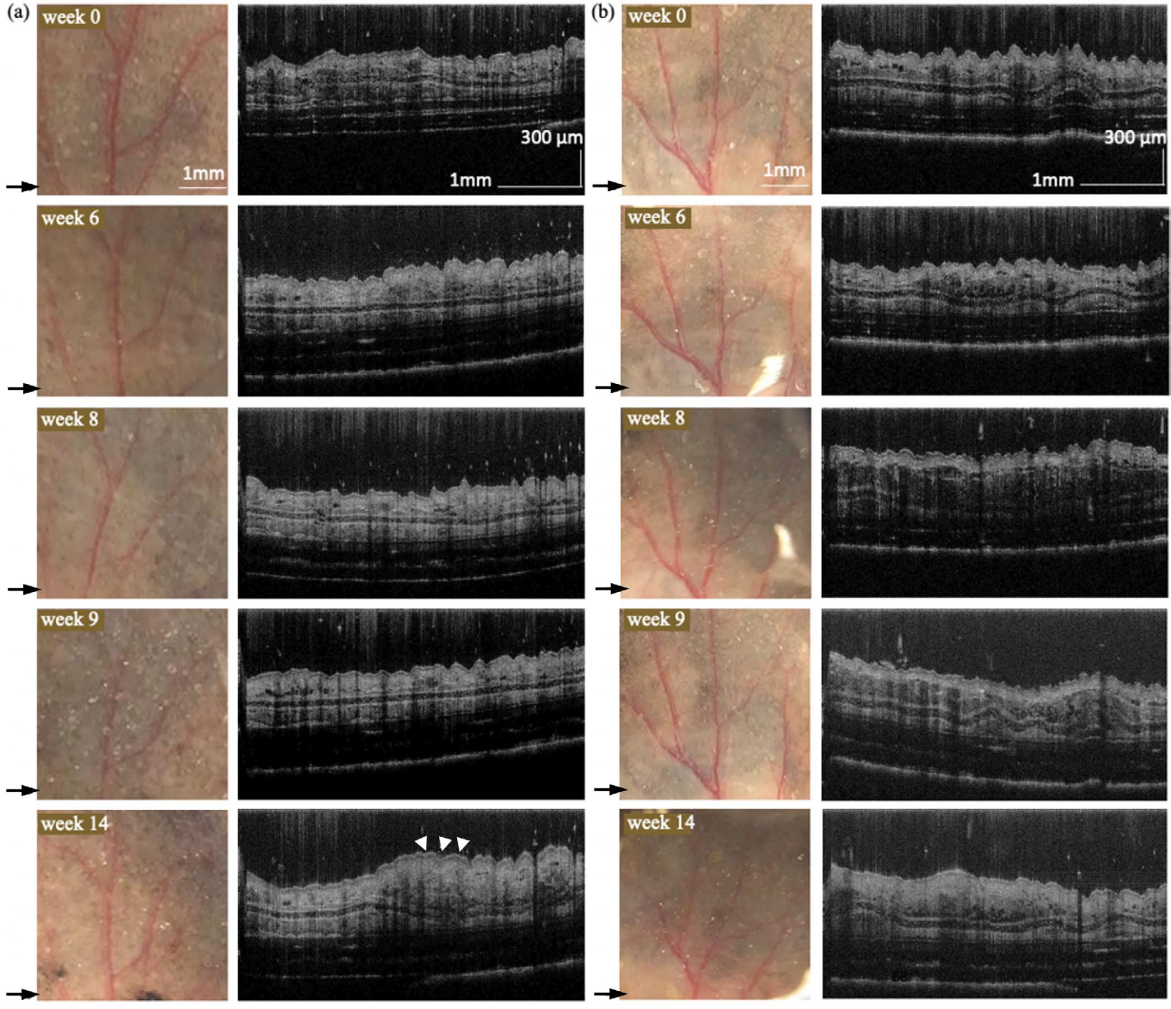
Photograph and corresponding cross‐sectional image of (a) dysplastic nevus and (b) control‐d mice at weeks 0, 6, 8, 9, and 14. Arrows indicate the location corresponding to the cross‐sectional image. White arrowheads indicate the increased skin thickness in the OCT cross‐sectional image of a dysplastic nevus mouse at week 14.

**FIGURE 7:**
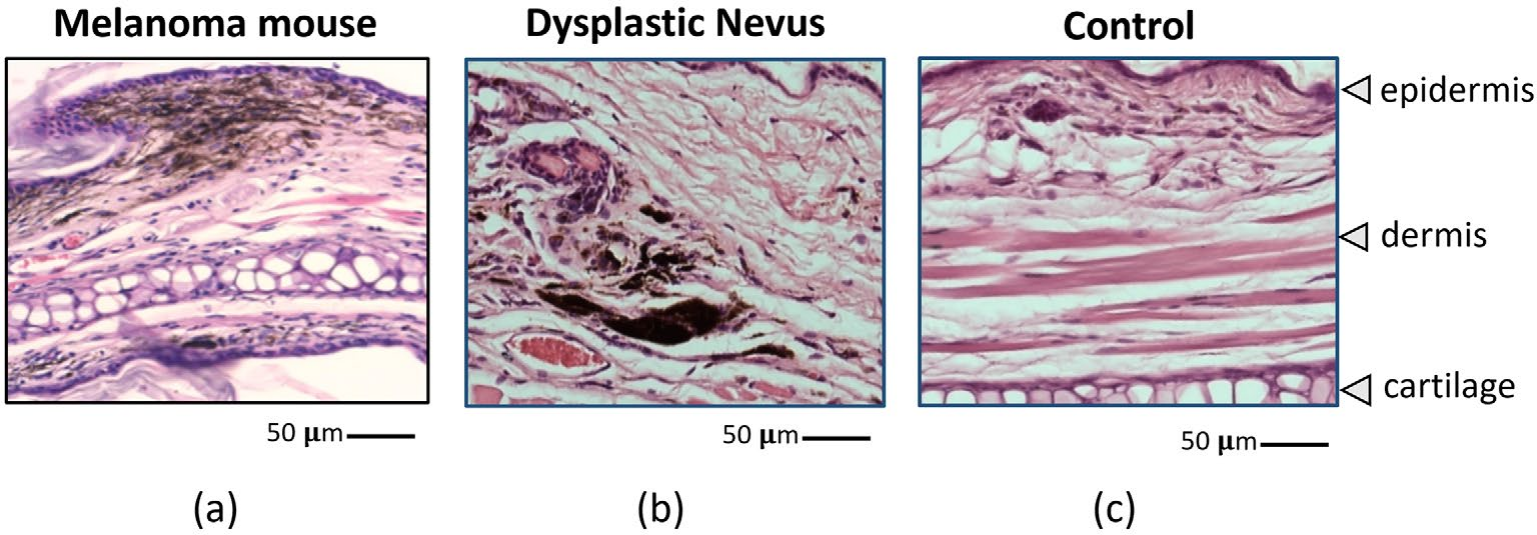
H&E section of (a) melanoma mouse at week 6, (b) dysplastic nevus mouse at week 14, and (c) control mouse at week 14.

Figure 6 presents photos and the related cross‐sectional image of dysplastic nevus (Figure 6a) and control‐d mice (Figure 6b) at various time points. Nevus formation on the skin occurs after 4‐HT initiation at week 6 and progressively grows more noticeable and compact. The OCT cross‐sectional image of the week 14 dysplastic nevus mouse shows increased skin thickness, marked by a white arrowhead. No specific features suggest increased malignancy with visual inspection until week 14. However, the melanoma probability score rises from weeks 8 to 9 and stays high after week 9 (Figure 5b). The μPET imaging was done from week 8 onwards to confirm the conversion of dysplastic nevi to malignant melanoma. Figure 5d displays the 18 F‐FDG accumulations in one representative dysplastic nevus mouse in weeks 8, 9, and 10. A tumor was detected on the head. Figure 7 shows H&E sections of melanoma mouse at week 6 (Figure 7a), dysplastic nevus mouse at week 14 (Figure 7b), and control mouse at week 14 (Figure 7c). Compared to control mice, melanoma and dysplastic nevus mice show accumulation of melanin. Pigmented cells spread throughout the dermis with loss of adipose tissue.

**FIGURE 8:**
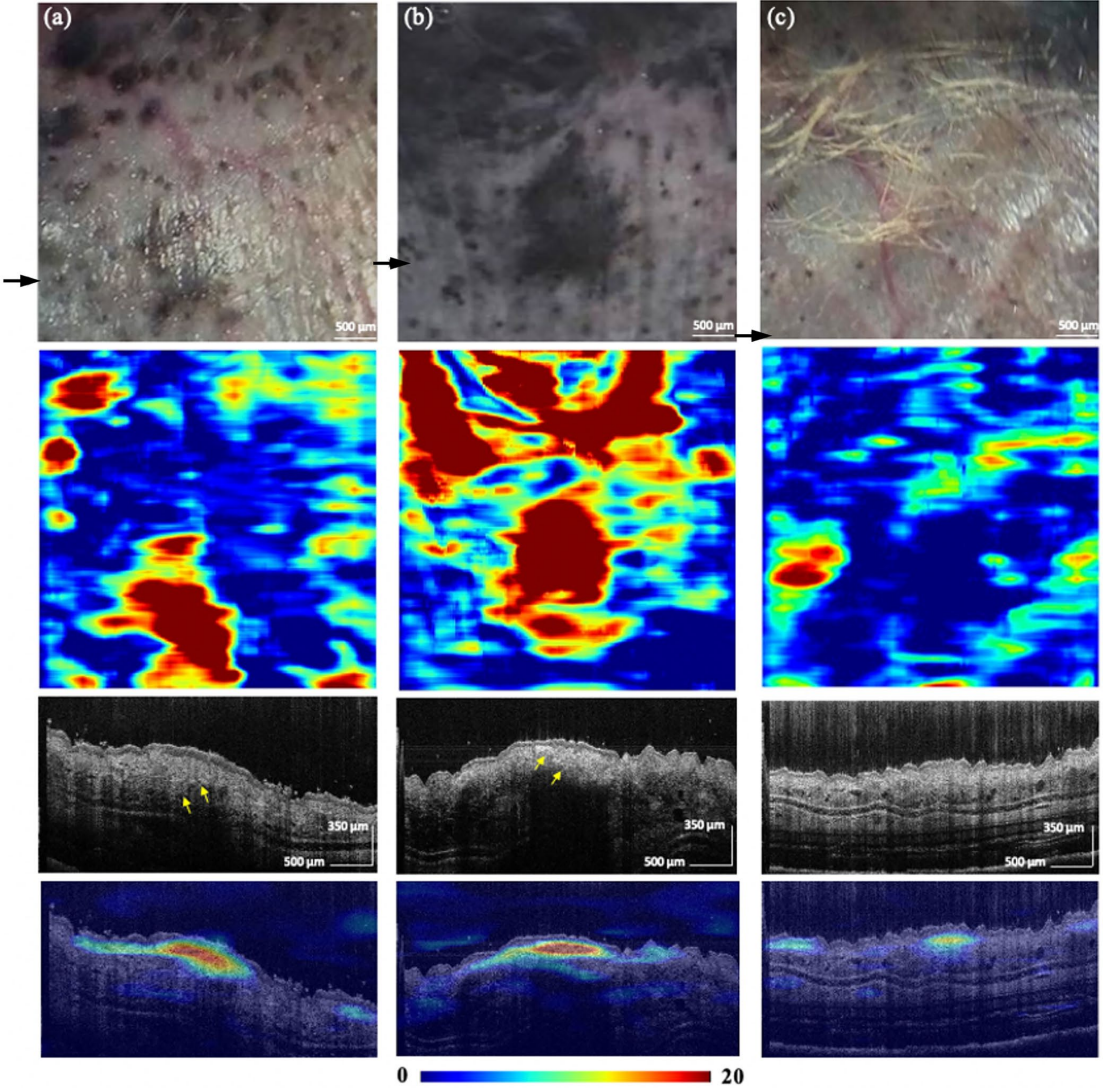
Photograph (the first row), en‐face projection of CAM (the second row), OCT cross‐sectional image (the third row), and OCT cross‐sectional image overlayed with CAM (the fourth row) of three melanomas with different sizes (a, b, c). Black arrows indicate the location corresponding to the OCT cross‐sectional image, whereas yellow arrows indicate a disarranged pattern in (a) and a hyperreflective region in (b).

We used a class activation map (CAM) analysis [26] to identify crucial regions for diagnosis. Figures 8a,b show that the CAM matches the tumor area in the photograph, indicating a meaningful decision. In the corresponding OCT image overlaid with CAM, the region receiving more attention from the DL model exhibits increased epidermis and dermis, architecture disarray, and hyperreflective structures. Figure 8c shows no noticeable melanoma‐specific features in this small melanoma (less than 1 mm). However, the CAM‐highlighted regions still matched the tumor in the photograph, and the area appeared brighter than the healthy region.

**FIGURE 9:**
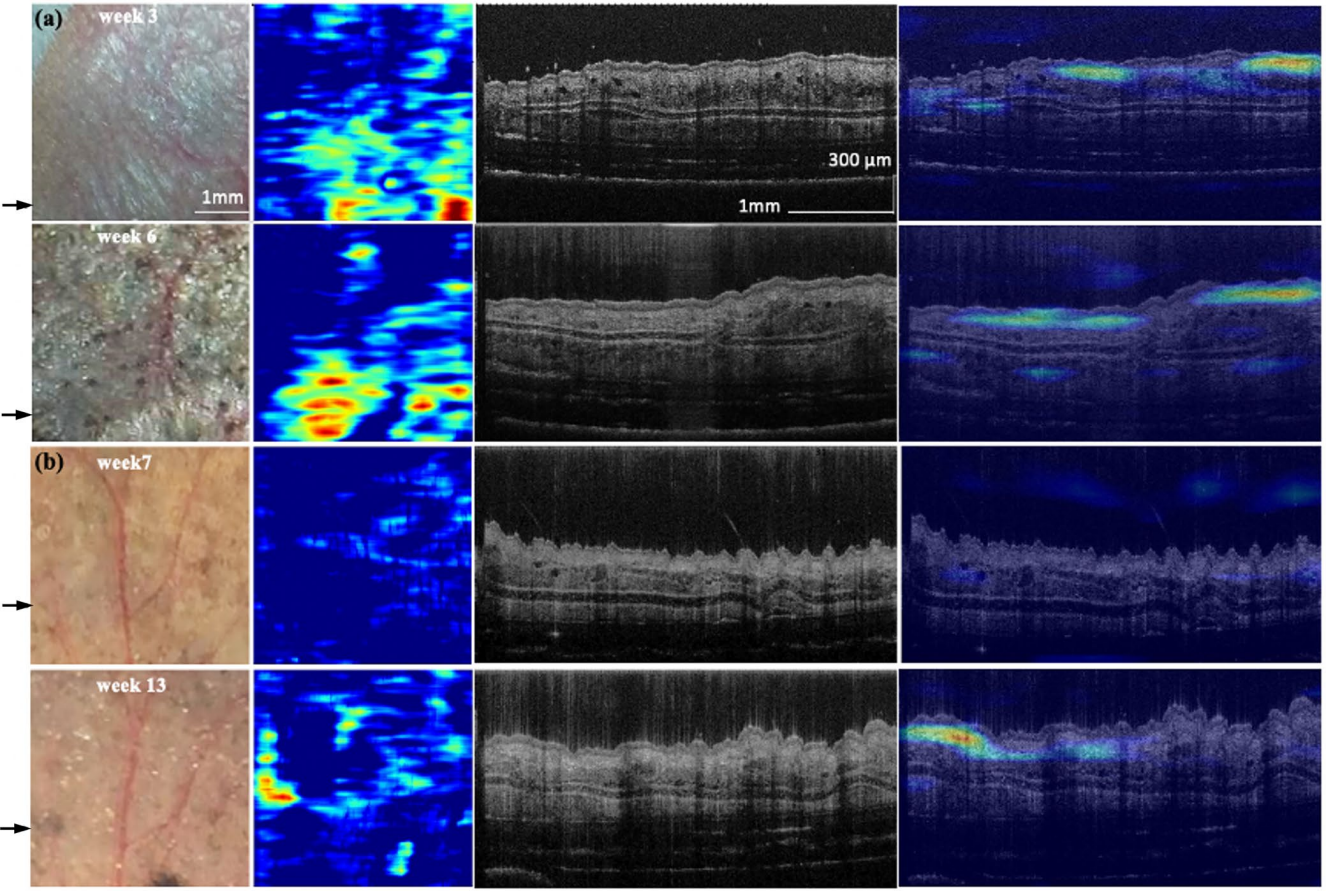
Photograph, en‐face projection of CAM, OCT cross‐sectional image, and OCT cross‐sectional image overlayed with CAM of (a) melanoma mice and (b) dysplastic nevus mice. Black arrows indicate the location corresponding to the OCT cross‐sectional image.

Furthermore, using the learned features, the trained model may be able to differentiate nevus from melanoma using the OCT cross‐sectional image. Figure 9a shows a photograph, en‐face maximum projection of CAM, OCT cross‐sectional image, and OCT cross‐sectional image overlayed with CAM of the same location in melanoma mice at weeks 3 and 6. Although there are only a few melanomas in the photograph, the trained model can perceive the abnormity in the OCT cross‐section image, and the region identified by the model is similar to melanoma in the photograph at week 6. On the contrary, although nevus appears on the skin at week 7, as shown in Figure 9b, no abnormality is detected in the CAM. However, after nevus gradually transformed into melanoma at week 13, the region highlighted by the CAM matches the photograph.

## 4 Discussion

This pioneering study integrates OCT with DL to continuously monitor melanoma progression in a specific animal model. Since this genetic mouse is driven by an oncogene commonly present in human cutaneous melanoma, it effectively mirrors human melanoma, offering a robust system for examining tumor development [22]. Although the thickness of mouse‐ear skin is less than that of human skin, it remains a pertinent and valuable model for investigating the development and detection of melanoma. First, mice have been used to induce and observe melanoma by UV exposure or genetic manipulation due to the similarity of the oncogene‐driven melanoma development between mice and humans [27, 28]. Second, mouse skin allows noninvasive and longitudinal imaging of melanoma progression and response to treatment using OCT [29]. Third, the mouse's ear skin has epidermal and dermal structures similar to human skin, and it is a convenient and widely used site. Consequently, our research using mouse models can offer valuable insights and demonstrate the potential utility of OCT and CNN in diagnosing human melanoma and predicting risks.

OCT is one of the emerging techniques that has been used to diagnose melanoma. Previous studies extract optical properties from cross‐sectional OCT images and use them to differentiate melanoma from benign lesions with great sensitivity of 93.3%–97% and a specificity of 96.7%–98% [13, 15, 16]. However, these reports were conducted on human subjects with large, visible lesions requiring excision. Besides, the optical characteristics are supposed to alter during tumor progression. In our dataset, the AC is insufficient to separate melanoma and control‐m mice (Figure 3), resulting in a poor sensitivity of 0.7 and specificity of 0.31. On the other hand, classification using radiomics features shows a performance improvement, having a sensitivity of 0.73 and a specificity of 0.63 is still unsatisfactory. This may be due to the relatively small diameter (< 1 mm) of most melanomas in our animal model compared to melanomas in human skin. Diagnosing melanomas with small diameters can be challenging. Hence, the algorithm we introduced in this study might prove helpful in detecting early‐stage cutaneous melanoma with lesions that are either too small or not visible to the naked eye. It could also help identify nevi likely to progress from benign lesions to melanoma. Accordingly, differences in study design and the subject model may explain the lower sensitivity and specificity observed in our AC and radiomics models compared to the studies referenced [13, 14, 15, 16].

Therefore, we utilized recent advancements in CNN technology, specifically VGG16, to categorize OCT cross‐sectional images of melanoma and control‐m mice. A sequential dataset was used, and the relative loss was introduced. This approach achieved a sensitivity of 0.99 and a specificity of 0.98 for classifying melanoma and healthy tissues. A previous review shows that computer‐aided diagnosis of melanoma using dermoscopic images has lower sensitivity and specificity than our method [30]. Another method uses a spatial–temporal network to compute the growth region and the melanoma probability scores for aligned lesion images over time [31]. However, aligning OCT images at different time points is difficult due to the μm scale. Instead, we compare the features extracted by VGG16 and ensure that the output score of the later time point is higher than the earlier one. After training, the model analyzes OCT cross‐sections of melanoma and control‐m mice from weeks 0 to 6 and dysplastic and control‐d mice from weeks 0 to 14. It is important to note that these images were not part of the training set. Assessing the same lesion at different time points shows that our model can consistently predict diagnostic results, with rapidly increasing probability scores in melanoma mice, relatively slower increases in dysplastic mice, and no apparent changes for control‐m and control‐d mice.

**FIGURE 10:**
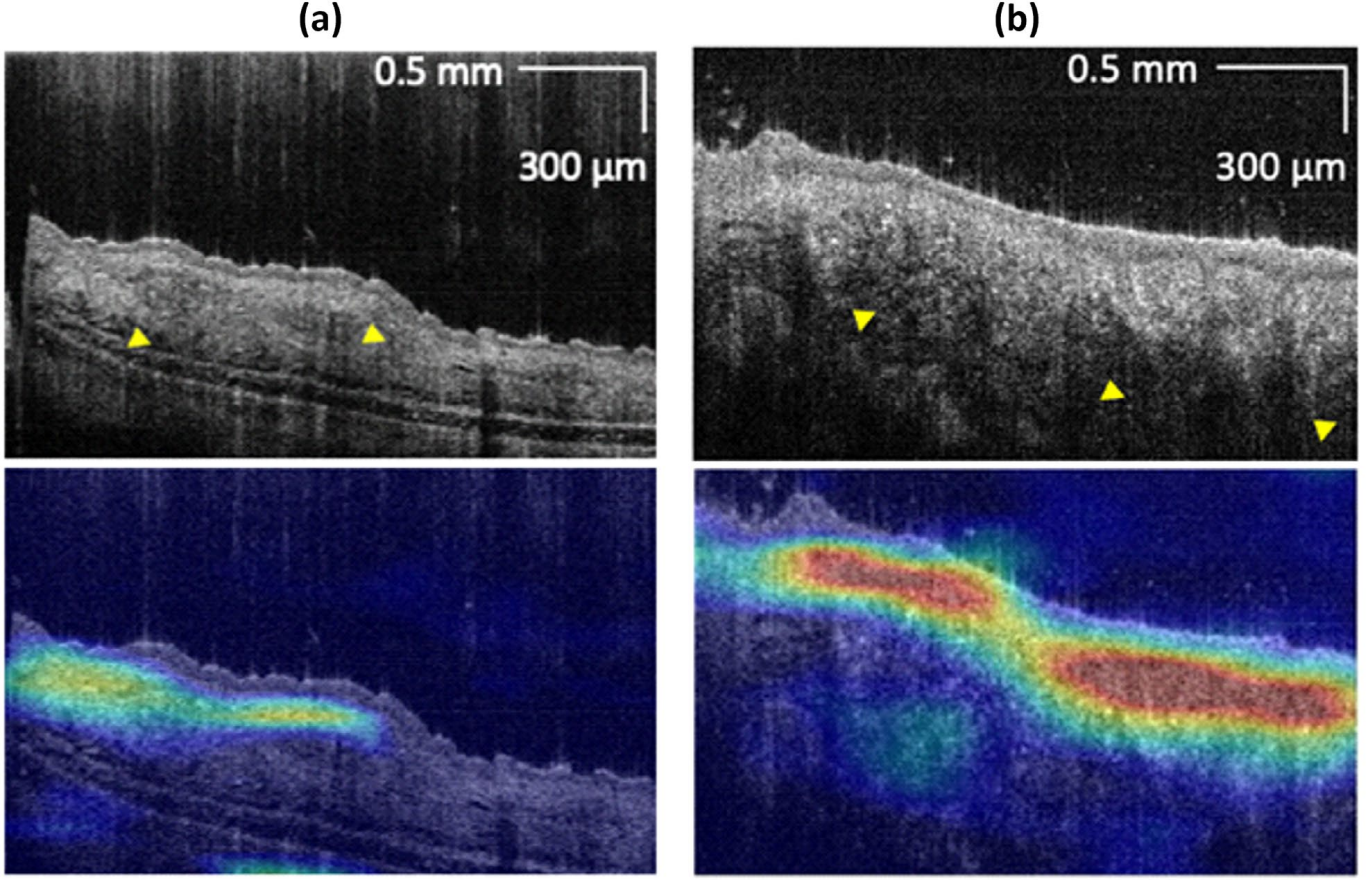
Patterns recognized by the deep learning model.

In this study, CAM is used to identify the most distinctive regions. The en‐face maximum projection of CAM resembles a photo of melanoma, suggesting the CNN network is making the correct decision. The regions marked by CAM show features of architectural disorder, less clear, and hyperreflective tissue, which agrees with previous work [11, 12, 13, 14]. Patterns automatically detected by CAM are similar to features defined by a specialist, as shown in Figure 10, where arrows indicate abundant reflective cytoplasm (Figure 10a) and icicle‐shaped structures (Figure 10b). Some regions marked by CAM do not show specific features that are easily visible to the naked eye. Therefore, we performed the statistical analysis, extracted radiomics features in these regions, and compared them with control‐m mice. The results show heterogeneity between the cancerous tissue and healthy tissues; the cancerous region has a higher intensity than healthy tissues, which aligns with previous findings [36].

However, artifacts from hair, gel bubbles, and out‐of‐focus areas in the OCT cross‐sectional images may affect the trained model's performance. This limitation can be overcome by removing hair and gel bubbles and improving tissue fixation beforehand. Another limitation of this study is the small number of mouse models used, which may not fully represent the diversity and heterogeneity of human skin and melanoma. Therefore, further validation of the proposed algorithm on human OCT images is necessary to evaluate its applicability and reliability in clinical settings. Additionally, a study with OCT images from different devices must be conducted to confirm the generalizability of the trained model.

## 5 Conclusions

Based on OCT sequential data, we are the first to use DL to differentiate melanoma from healthy tissues over time. We expect this algorithm to capture the essential features and changes of melanoma over time and provide helpful information for diagnosis and prognosis. Since the size of melanoma in humans is more significant than that in mice, leading to more prominent melanoma‐specific characteristics in human skin tissue and more accurate classification results. Future work will optimize and validate the CNN model on a more diverse dataset. Melanoma probability scores provided by the proposed algorithm may also help identify high‐risk nevi with a higher chance of malignant transformation.

## Author Contributions

P.‐Y.L.: data acquisition, analysis, and interpretation; writing – original draft preparation. T.‐Y.S.: data acquisition, analysis, and interpretation. Y.H.‐C.: data acquisition and analysis. C.‐H.C.: conceptualization, methodology, resources. W.C.‐K.: conceptualization, methodology, supervision, funding acquisition, writing – reviewing and editing.

## Ethics Statement

All animal experiments were performed according to the Institutional Animal Care and Use Committee of National Yang Ming Chiao Tung University guidelines and approved by the committee (IACUC approval no. 1071201).

## Conflicts of Interest

The authors declare no conflicts of interest.

## Data Availability

The data that support the findings of this study are available from the corresponding author upon reasonable request.
